# Effects of cooking using multi-ply cookware on absorption of potassium and vitamins: a randomized double-blind placebo control study

**DOI:** 10.3109/09637486.2011.642342

**Published:** 2012-01-09

**Authors:** Mari Mori, Atsumi Hamada, Hideki Mori, Yukio Yamori, Kinsuke Tsuda

**Affiliations:** 1 Mukogawa Women's University Institute for World Health Development, Edagawa-cho, Nishinomiya-shi, Hyogo, Japan; 2 St Agnes’ University Japan Institute Food Education and Health, Gomen-cho, Kamigyo-ku, Kyoto, Japan; 3 Graduate School of Human and Environmental Studies, Kyoto University, Yoshida-nihonmatsu-cho, Sakyou-ku, Kyoto, Japan

**Keywords:** vegetable ingestion, water-free cooking, 24-h urinary NalK, vitamin C, β-carotene, oxidized LDL

## Abstract

This 2-week interventional study involved a randomized allocation of subjects into three groups: Group A (daily ingestion of 350 g vegetables cooked without water using multi-ply [multilayer-structured] cookware), Group B (daily ingestion of 350g vegetables; ordinary cookware) and Group C (routine living). Before and after intervention, each subject underwent health examination with 24-h urine sampling. Blood vitamin C significantly increased after intervention from the baseline in Group A (*P* < 0.01) and Group B (*P* < 0.05). β-Carotene levels also increased significantly after intervention in Group A (*P* < 0.01) and Group B (*P* < 0.01). Oxidized low-density lipoprotein decreased significantly after intervention in Group A (*P* < 0.01). In Group A, 24-h urinary potassium excretion increased significantly (*P* < 0.01) and 24-h urinary sodium (Na)/K ratio improved significantly (*P* < 0.05) after intervention. In conclusion, a cooking method modification with multi-ply cookware improved absorption of nutrients from vegetables and enhanced effective utilization of the antioxidant potentials of vegetable nutrients.

## Introduction

Ingestion of vegetables in the daily diet prevents lifestyle-related diseases (Hooper 2001). In Japan, the National Health Promotion Campaigns for the 21^st^ Century (Healthy Japan 21), advocated by the Ministry of Health, Labour and Welfare (2000), recommend adults ingest 350 g or more vegetables per day for maintenance of good health. In practice, however, the amount of vegetables eaten daily by individuals aged between 20 and 39 years is approxi- mately 250 g, which is below the target level (Ministry of Health, Labour and Welfare 2009). The goal of ingesting 350 g or more of vegetables per day can be achieved if vegetables eaten are cooked (rather than raw) and if the amount required daily is divided into three meals. However, individuals, particularly stu- dents, increasingly skip breakfast or rely on fast food as one of their three meals (Osako et al. 2005). For these individuals, daily ingestion of 350 g vegetables is difficult to achieve, and some investigators reportedthat young men and women consumed an average of 130g of vegetables per day. Additionally, the use of water to cook vegetables should be avoided as far as possible so that the nutrients, which are likely to be eluted into water, are preserved (Kimura and Itokawa 1990; Agte et al. 2002; Gupta and Bains 2006). The purpose of the present study was to examine whether the absorption of vegetable nutrients is higher when vegetables are cooked using traditional methods or using modified cookware to preserve water-soluble or heat-sensitive nutrients. We asked the randomized volunteers to use modified cookware that enabled more efficient nutrient intake rather than ingesting vegetables cooked by ordinary cookware, and analyzed changes in the levels of 24-hour urine sample (24 U) K excretion, blood vitamins and oxidized low-density lipoprotein cholesterol (ox-LDL), among other values.

## Methods

### Subjects

Of the undergraduate and graduate students of Kyoto University, those living alone and capable of cooking at home were invited to an orientation meeting during which the study design and details were explained. The criteria of exclusion were (1) the student who was not living alone, (2) the student who disliked vegetables and (3) the student living in a room without a kitchen. And 90 students understood the study design and details and provided informed consent after the orientation, and were enrolled in the study. All subjects underwent health examination before the start of the study, and subjects were randomly divided into three groups: Group A, daily ingestion of 350 g vegetables cooked without water using a ‘multi-ply cookware’ (fully multilayer-structured cookware #5123, Vita Craft Japan Ltd, Kobe, Japan); Group B, daily ingestion of 350 g vegetables cooked using an ordinary cookware and Group C, ordinary daily living. This study was performed under ethical considerations in compliance with ‘The Declaration of Helsinki - Ethical Principles for Medical Research Involving Human Subjects’ and ‘The Ethical Guidelines on Epidemiological Studies’ (Ministry of Education, Culture, Sports, Science, and Technology and Ministry of Health, Labour and Welfare). During the study, we took adequate care of the life, health and privacy of individual contributors, staff members and subjects. Informed consent was obtained from each subject before the start of the study. This study was approved in advance by the Ethics Committee of the Kyoto University Graduate School of Human and Environmental Studies and the Ethics Committee of Mukogawa Women's University.

### Schedule

**Figure 1:**
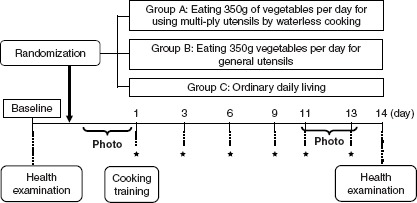
Design of the study. An initial screening visit was conducted 2 weeks prior to enrolment and randomization for 3 groups. Blood samples were collected before and after the periods of vegetable intake. Photo: Photos of all diets taken for 3 days; *: vegetable delivery.

Of the subjects randomly allocated to the three groups, those allocated to Groups A and B (groups to which cookware was provided) received cooking training (separate training for each of these two groups) so that the subjects would use the provided cookware appropriately. Next, the apparently similar cookware (one unit) was provided to individual subjects in Groups A and B. The cookware and first set of vegetables were supplied simultaneously, and the subjects began to consume the supplied vegetables on the day of allocation. To retain freshness of the supplied vegetables, vegetables were supplied twice per week (six supplies during the study period). Amounts of unused vegetables and discarded amounts were recorded; full ingestion of supplied vegetables was preferred. Health examinations, identical to those performed before the start of the study, were performed 2 weeks after the start of vegetable consumption (Figure 1).

### Test diet

To ensure that a uniform amount of vegetables was ingested daily by each subject, consistent amounts of vegetables were supplied at six time points during the study period. Table I lists the vegetables supplied during the study.

**Table I:** List of vegetables supplied during the study.

1–3 Days		4∼7 Days		8∼11 Days		12∼14 Days	
			
Item	g	Item	g	Item	g	Item	g
Spinach	400	Pumpkin	200	Komatsuna	150	Okra	100
Cabbage	500	Kidney bean	50	Okra	100	Broccoli	100
Green asparagus	100	Carrot	200	Broccoli	100	Potatoes	150
Carrot	50	Green asparagus	200	Bean sprouts	450	Komatsuna	300
Garlic	10	Paprika	110	Leek	50	Lotus	50
		Grape tomato	90	Garlic	20	Jew's mallow	250
		Spinach	150	Cabbage	500	Yam	100
		Bean sprouts	100	Pepper	50		
		Garlic	10				
		Potatoes	200				
		Onion	100				

Notes: The weight including vegetable skin. Participants weregiventoeat 350 gofvegetables per day.

### Measurement at the time of health examination and during the study period

Health examination before and after the study included the measurement of height, body weight, body fat ratio, blood pressure, heart rate (using an automated blood pressure measurement system; HEM-907 Omron, Kyoto, Japan), fasting blood sampling and by 24 U collection using an aliquot cup. The photography of meals before dieting and diary tracking (to record supplied vegetable consumption) were collected during health examination before and/or after the study. In each blood sample, we measured peripheral blood parameters (white and red blood cell counts [WBC and RBC], haemoglobin levels, haematocrit levels, mean corpuscular volume [MCV], mean corpuscular haemoglobin [MCH] level, MCH concentration and platelet count) and the levels of total cholesterol, high-density lipoprotein cholesterol (HDL), LDL cholesterol, ox-LDL by ELISA method, triglycerides, folic acid, homocysteine, blood glucose, haemoglobin Ale, aspartate aminotransferase, alanine aminotransferase, γ-glutamyl transpeptidase, vitamin C, β-carotene and insulin. The levels of Na, K, creatinine and 8-hydroxydeoxyguanosin were measured in the 24 U sample. Photographs taken before taking meals were used to assess the amount of vegetables consumed before and after the start of the study. Photographs of all vegetables consumed over a 3-day period before the start of the study and for 3 days before the completion of the vegetable consumption period (6 days in total) were acquired using a mobile phone camera by individual subjects. Photographs taken were saved on a recording medium for mobile phones (microSD) supplied by the researchers and collected at the time of the last health examination. Each subject maintained a diary to record the vegetables consumed relative to those supplied, the vegetables discarded without being consumed, physi- cal condition and supplement intake, as well as other relevant factors, every day during the study period.

### Adverse events

One subject withdrew from participation in the study after the pre-study health examination and before randomized allocation for a personal reason not related to the study design. One subject from Group A quit the study because of fever onset on the day of post-study health examination. One subject quit the study on the day of the post-study health examination for a personal reason not related to the study design.

All of these events were judged to have no association with the test diet according to the principal investigator and the physician responsible at the testing facility.

### Statistical analysis

**Figure 2:**
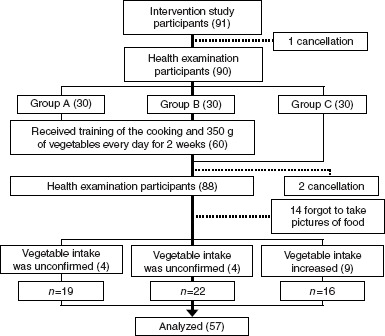
Trial profile.

A third party who was not part of the study staff evaluated the amounts of vegetables consumed by individual subjects using the photographs of meals taken over a 6-day period before and after the start of the study. Analysis included 57 subjects, and subjects each in Groups A and B to whom vegetables were supplied increased vegetable consumption and sub- jects in Group C did not increase the amount of vegetables ingested. Analysis of the data obtained from 24 U analysis included 45 subjects (16 from Group A, 15 from Group B and 14 from Group C) in whom 24 U collection was objectively rated as successful on the basis of self-reports and urinary creatinine levels (Figure 2). The health examination data before and after the start of the study in these subjects were evaluated using the *t*-test. The magnitude of change after the start of the study was compared among the three groups using analysis of variance. Statistical analysis was performed using SPSS Ver. 15.0. *P* < 0.05 was regarded as statistically significant.

## Results

### Comparison before and after vegetable intake

Blood vitamin C levels increased significantly after the study as compared with the baseline levels before the study in Group A (9.2 ± 1.6 → 11.0 ± 1.7 μg/ml; *P*<0.01) and Group B (9.8 + 2.4 → 10.7 + 2.0 μg/ml; *P*<0.05), while no significant change was observed in Group C. Blood β-carotene level increased significantly after the study in Group A (59.8 ± 45.8 → 74.4 ± 40.2 μg/dl; *P*<0.01) and Group B (43.4 + 48.9 → 60.3 + 43.9 jig/dl; *P* < 0.01), while there was no statistically significant change in Group C. Blood folic acid level generally increased after the study in Group A (5.6± 1.6 → 6.1 ± 1.7ng/ml; *P*=0.07) and increased significantly after the study in Group B (5.6 ± 1.8 → 6.4 ± 2.2ng/ml; *P* = 0.05), while there was no statistically significant change in Group C. Blood uric acid level generally decreased after the study in Group A (4.9 ± 0.8 → 4.6 ± 0.8 mg/dl; *P*=0.06), while no change was observed in Group B or C. Blood total cholesterol level decreased significantly after the study in Group A (187.2 ± 39.0 → 168.8 ± 27.6mg/dl; *P* < 0.01) and Group B (179.7 ± 22.5 → 163.2 ± 30.4 mg/dl; *P*<0.01), while no statistically significant change was observed in Group C. Blood LDL level decreased significantly after the study in Group A (99.5 ± 31.9 → 89.6 ± 23.6mg/dl; *P*<0.05) and Group B (98.3 ± 29.8 → 87.8 ± 23.1 mg/dl; *P*< 0.01), but no statistically significant change was observed in Group C. Blood ox-LDL level decreased significantly after the study in Group A (101.5 ± 31.9 → 84.1 ± 19.7U/l; *P*<0.01), while it showed no significant change in Group B or C (Table II).

**Table II:** Comparison between the baseline and the data after vegetable intake.

	Group A (*n* 19 = 8:11)		Group B (*n* 22 = 8:14)		Group C (*n* 16 = 4:12)	
			
	Baseline	2 Weeks	Baseline	2 Weeks	Baseline	2 Weeks
Age	20.4 ± 2.4		20.2 ± 1.9		20.9 ± 2.9	
Body fat(%)	21.9 ± 7.7	20.5 ± 7.7**	24.6 ± 7.1	22.9 ± 6.4**	25.4 ± 6.0	24.0 ± 5.4**
BMI (kg/m^2^)	20.7 ± 2.2	20.7 ± 2.3	21.1 ± 2.0	20.9 ± 2.0	20.5 ± 1.8	20.5 ± 1.9
Systolic BP (mm Hg)	112.6 ± 13.8	110.9 ± 13.0	112.2 ± 8.0	111.4 ± 6.9	108.6 ± 10.3	101.8 ± 26.7
Diastolic BP (mm Hg)	62.8 ± 6.7	60.9 ± 7.6	62.2 ± 6.6	61.5 ± 6.4	62.4 ± 7.1	60.9 ± 7.2
Heartrate (bpm)	69.9 ± 11.7	67.1 ± 10.1	70.5 ± 7.1	72.4 ± 8.7	68.9 ± 10.9	68.4 ± 10.0
ALT(GPT) (U/l)	15.4 ± 5.9	14.4 ± 6.7	16.7 ± 10.4	12.6 ± 5.7**	14.8 ± 4.2	13.8 ± 4.4
AST (GOT) (U/l)	20.3 ± 5.3	19.5 ± 4.7	22.2 ± 14.7	18.0 ± 5.0	19.6 ± 4.8	19.3 ± 3.4
HDL (mg/dl)	71.1 ± 16.0	67.3 ± 16.8*	67.0 ± 15.8	62.4 ± 13.7*	70.9 ± 16.5	68.8 ± 11.3
LDL (mg/dl)	99.5 ± 31.9	89.6 ± 23.6*	98.3 ± 29.8	87.8 ± 23.1**	93.3 ± 26.6	94.0 ± 23.7
β-Carotene (mg/dl)	59.8 ± 45.8	74.4 ± 40.2**	43.4 ± 48.9	60.3 ± 43.9**	50.6 ± 33.6	48.0 ± 31.0
γ-GTP (U/l)	14.8 ± 3.4	14.1 ± 3.2	12.9 ± 5.0	12.5 ± 5.0	17.0 ± 5.9	16.4 ± 6.5
Insulin (μIU/ml)	5.4 ± 3.1	6.5 ± 5.7	6.7 ± 2.8	8.9 ± 8.5	8.3 ± 5.4	6.9 ± 4.1
Glucose (μg/dl)	91.5 ± 6.5	89.8 ± 6.9	94.0 ± 5.6	92.2 ± 8.5	91.1 ± 5.6	86.8 ± 7.3
HOMA-R	1.2 ± 0.8	1.5 ± 1.3	1.6 ± 0.7	2.1 ± 2.3	1.9 ± 1.4	1.5 ± 0.8
Vitamin C(μg/ml)	9.2 ± 1.6	11.0 ± 1.7**	9.8 ± 2.4	10.7 ± 2.0*	10.2 ± 2.1	9.9 ± 2.2
Ox-LDL (U/l)	101.5 ± 31.9	84.1 ± 19.7**	96.1 ± 25.3	88.2 ± 27.5	104.3 ± 65.0	89.1 ± 33.5
Total-C (mg/dl)	187.2 ± 39.0	168.8 ± 27.6**	179.7 ± 33.5	163.2 ± 30.4**	182.9 ± 38.0	177.1 ± 28.2
Total Hcys (nmol/ml)	8.5 ± 1.8	8.7 ± 1.9	11.5 ± 8.0	12.9 ± 12.7	9.1 ± 2.6	8.4 ± 2.0
Triglyceride (mg/dl)	50.0 ± 20.3	55.4 ± 23.2	57.7 ± 34.8	62.9 ± 52.8	57.6 ± 22.8	52.5 ± 21.7
Uric acid (mg/dl)	4.9 ± 0.8	4.6 ± 0.8	5.2 ± 0.7	5.1 ± 1.0	5.1 ± 0.9	5.0 ± 1.3
Folic acid (ng/ml)	5.6 ± 1.6	6.1 ± 1.7	5.6 ± 1.8	6.4 ± 2.2	6.0 ± 2.1	6.4 ± 2.4
Hs-CRP (ng/ml)	250.6 ± 447.6	292.0 ± 392.9	433.0 ± 1044.1	271.0 ± 448.7	422.6 ± 769.2	1489.9 ± 4691.1

Notes: *n* = men: women. Values are means ± SD. HOMA-R: insulin resistance = insulin × glucose/405. Ox-LDL, oxidized LDL; Total-C, total cholesterol; Total Hcys, total homocysteine; Hs-CRP, high-sensitive C-reactive protein. Significantly different from the baseline (

* *P* < 0.05,

** *P* < 0.01, two-side paired *t*-test).

### Magnitude of change between the vitamin levels before and after vegetable intake

The magnitude of change in vitamin C levels before and after vegetable intake in Groups A was significantly different (*P*<0.05) from Group C. The magnitude of change in β-carotene level was significantly different between Groups A and C (*P* < 0.05) and between Groups B and C (*P* < 0.01; Table III).

**Table III:** Change from the baseline to the data after vegetable intake.

	Group A (*n* 19 = 8:11)	Group B (*n* 22 = 8:14)	Group C(*n* 16 = 4:12)
Body fat(%)	− 1.41 ± 1.81	− 1.67 ± 1.07	− 1.34 ± 1.71
BMI (kg/m^2^)	− 0.06 ± 0.47	− 0.12 ± 0.31	− 0.08 ± 0.45
Systolic BP (mm Hg)	− 1.68 ± 7.75	− 0.82 ± 7.79	− 6.88 ± 25.84
Diastolic BP (mm Hg)	− 1.95 ± 7.43	− 0.68 ± 5.38	− 1.56 ± 6.11
Heartrate (bpm)	− 2.84 ± 10.19	1.86 ± 7.19	− 0.44 ± 8.97
ALT(U/l)	− 1.05 ± 5.18	− 4.05 ± 6.35	− 0.94 ± 4.77
AST (U/l)	− 0.84 ± 4.56	− 4.23 ± 12.63	− 0.25 ± 2.70
HDL-C (mg/dl)	− 3.84 ± 7.49	− 4.55 ± 10.03	− 2.13 ± 7.72
LDL-C (mg/dl)	− 9.89 ± 17.35	− 10.55 ± 14.99	0.69 ± 12.77
β-Carotene (mg/dl)	14.55 ± 21.82†	16.90 ± 15.76††	− 2.59 ± 17.21
γ-GTP (U/l)	− 0.79 ± 2.12	− 0.36 ± 1.33	− 0.63 ± 1.67
Insulin (μIU/ml)	1.16 ± 3.39	2.16 ± 7.54	− 1.43 ± 6.53
Glucose (mg/dl)	− 1.63 ± 6.09	− 1.77 ± 8.12	− 4.25 ± 8.79
HOMA-R	0.25 ± 0.78	0.56 ± 2.08	− 0.45 ± 1.63
Vitamin C(μg/ml)	1.81 ± 1.79†	0.93 ± 2.00	− 0.32 ± 2.96
Ox-LDL (U/l)	− 17.42 ± 25.18	− 7.86 ± 29.82	215.19 ± 58.72
Total-C (mg/dl)	− 18.37 ± 23.96	− 16.55 ± 17.84	− 5.81 ± 19.15
Total Hcys (nmol/ml)	0.23 ± 0.95	1.49 ± 5.11	− 0.67 ± 1.31
Triglyceride (mg/dl)	5.37 ± 27.60	5.23 ± 48.78	− 5.13 ± 14.40
Uric acid (mg/dl)	− 0.26 ± 0.56	− 0.10 ± 0.55	− 0.09 ± 0.76
Folic acid (ng/ml)	0.50 ± 1.12	0.77 ± 1.76	0.34 ± 1.76
Hs-CRP (ng/ml)	41.42 ± 353.57	− 162.00 ± 1178.16	1067.21 ± 4824.67

Notes: *n* = men:women. Values are means ± SD. HOMA-R: insulin resistance = insulin × glucose/405. ox-LDL, oxidized LDL; Total-C, total cholesterol; Total Hcys, total homocysteine; Hs-CRP, high-sensitive C-reactive protein. Significantly different from Group C at week 2 (

† : *P* < 0.05

†† : *P* < 0.01).

### Results of 24 U sample test

The amount of K excreted daily into urine (calculated as K) increased significantly after the study compared with the levels before the study in Group A (1.6 ± 0.9 → 2.0 ± 1.1 g; *P*< 0.01), while it showed no statistically significant changes in Group B or C. The Na/K ratio decreased significantly in Group A (3.7 ± 2.0 → 2.6 ± 0.9; *P*< 0.05), while no statistically significant changes were observed in Group B or C (Table IV).

**Table IV:** Comparison between the baseline and the 24 U data after vegetable intake.

	Group A (*n* 16 = 7:9)		Group B (*n* 15 = 4:11)		Group C (*n* 14 = 3:11)	
			
Urine	Baseline	2 Weeks	Baseline	2 Weeks	Baseline	2 Weeks
8-OHdG (μg/day)	3.7 ± 2.1	3.8 ± 2.0	3.5 ± 1.4	3.9 ± 1.6	4.6 ± 1.4	3.9 ± 1.7
Na/K	3.7 ± 2.0	2.6 ± 0.9*	3.8 ± 1.7	3.1 ± 1.6	3.2 ± 1.2	3.5 ± 2.0
NaCl (g/day)	7.8 ± 4.2	7.7 ± 4.0	8.2 ± 4.0	7.4 ± 3.2	7.2 ± 2.3	7.3 ± 2.7
K(g/day)	1.6 ± 0.9	2.0 ± 1.1**	1.5 ± 0.5	1.6 ± 0.4	1.6 ± 0.6	1.6 ± 0.8

Notes: n = men:women. Values are means ± SD. 8-OhdG, 8-hydroxy-20-deoxyguanosine. Significantly different from the baseline (

* *P* < 0.05,

** *P* < 0.01, two-side paired *t*-test). The analysis only as for the success in 24 U collection.

## Discussion

The present study was designed to evaluate the differential effect on the absorption of vegetable nutrients by using two cookware and to objectively analyze the difference by examining blood and 24 U biomarkers of nutrients. Cooking using multiply cookware, designated as Group A, was characterized as water-free cooking, including boiling vegetables using only the moisture contained in the vegetables. Ordinary heating or boiling of vegetables using water is likely to result in the elution of a high percentage of vitamins and minerals in the vegetables (Kimura and Itokawa 1990) and these nutrients are decreased in ready-to-eat foods (Agte et al. 2002) compared with traditional foods (Gupta and Bains 2006), while water-free cooking minimizes the elution of nutrients, enabling efficient intake of vegetable nutrients (Gordon and Noble 1964). K levels in 24 U samples of Groups A and B rose significantly after intervention, suggesting that the nutrients contained in vegetables supplied as the test diet (350 g daily) had been consumed by the subjects since previous studies reported that 24 U K was a good biomarker of vegetable consumption (Tuekpe et al. 2006; Mente et al. 2009). Particularly water-free cooking resulted in a significant increase in 24 U K excretion, indicating K was absorbed efficiently because of no loss of K into water during cooking. This 24 U K increase was not accompanied with increase in 24 U Na excretion, and 24 U Na/K ratio was significantly reduced in Group A. Therefore, participants in Group A were supposed to have enjoyed the natural taste of vegetables themselves without adding salt for seasoning. This was confirmed by questionnaires after the study (not published). WHO-coordinated CARDIAC study covering 61 populations in 25 countries proved that 24 U Na and Na/K ratio were inversely related to stroke mortality ratio in the world (Yamori et al. 2006), indicating that increased K intake and decreased Na/K ratio concomitantly observed particularly in Group A may contribute to the possible risk reduction of stroke. Moreover, serum total and LDL cholesterol observed in Groups A and B taking more vegetables confirmed the well-known benefit effect of vegetable intake (Djousse et al. 2004) and the related risk reduction of coronary heart diseases (Yamori et al. 2006). Blood vitamin C and β-carotene levels also increased significantly after intervention in Group A, accompanied by a significant reduction in blood ox-LDL level. There is a report which clearly demonstrated that vitamin C prevented increased endothelial permeability caused by ox-LDL (May and Qu 2010). Increased endothelial permeability results in the development of atherosclerosis vascular lesions (Fujita et al. 2009). Therefore, vitamin C elevation and concomitant ox-LDL reduction observed in Group A indicate that the ingestion of vegetables cooked by the modified cookware is expected to reduce the risk of cardiovascular diseases and major outcome of atherosclerotic vascular lesions in the long run.

## Conclusions

This randomized double-blind study comparing the intake of similar amounts of provided vegetables cooked with water-free efficient cookware with the intake of vegetables cooked with ordinary cookware (placebo) has confirmed that using this water-free cooking method enables more efficient utilization of the antioxidant potential of vegetables, which is expected to have better effects on cardiovascular risk reduction than ingesting the vegetables cooked by ordinary methods.

## Affiliations

All authors were involved in the concept and design of this study. AH and HM collected the data and performed the nutritional analyses. YY and KT assessed the quality of study design and the data independently. MM performed the statistical analysis and wrote the manuscript. All authors critically reviewed the manuscript and approved the final version submitted for publication.
